# Assessment of Eyelid Pressure Using a Novel Pressure Measurement Device in Patients With Moderate-to-Severe Dry Eye Disease

**DOI:** 10.3389/fmed.2022.833576

**Published:** 2022-02-07

**Authors:** Jingyi Wang, Jiayu Bao, Wenxiu Song, Siyuan Li, Yiran Hao, Lei Tian, Ying Jie

**Affiliations:** ^1^Beijing Ophthalmology and Visual Sciences Key Laboratory, Beijing Tongren Eye Center, Beijing Institute of Ophthalmology, Beijing Tongren Hospital, Capital Medical University, Beijing, China; ^2^Beijing Aier Intech Eye Hospital, Beijing, China; ^3^Beijing Advanced Innovation Center for Big Data-Based Precision Medicine, Beihang University and Capital Medical University, Beijing, China

**Keywords:** eyelid pressure, friction, tear film, ocular surface parameters, moderate-to-severe dry eye disease

## Abstract

**Objective:**

To assess a novel eyelid pressure measurement device and study the relationship between eyelid pressure and ocular surface parameters of moderate-to-severe dry eye disease (DED).

**Methods:**

The present study included 70 eyes of 35 moderate-to-severe DED patients. All subjects were subjected to the following examinations for DED assessment: Ocular Surface Disease Index (OSDI) questionnaire, tear meniscus height (TMH), lipid layer thickness (LLT), number of partial blink (PB), total blink (TB) and partial blink rate (PBR), fluorescein tear breakup time (FBUT), corneal fluorescein staining (CFS), lid margin abnormality, meibum expression assessment (meibum score), meibomian gland dropout (MGd) and Schirmer I test. Pressure of the upper eyelid was measured thrice with the novel pressure measurement device. Repeatability of the device was evaluated by intraclass correlation coefficient (ICC). Safety of the device was evaluated by observing ocular adverse reactions of each subject prior to measurement, at day 1 and day 7 following measurement. Correlations between eyelid pressure and ocular surface parameters of moderate-to-severe DED were analyzed by using Pearson correlation coefficient and Kendall's tau-b correlation coefficient.

**Results:**

ICC of the measurement results in our study was 0.86. There was no abnormality presenting in all subjects recorded prior to measurement, 1 and 7 days following measurement. The eyelid pressure was significantly correlated with PBR (r = 0.286, *P* = 0.016), FBUT (r = −0.331, *P* = 0.005), CFS (r = 0.528, *P* = 0.000), lid margin abnormality (r = 0.408, *P* = 0.011) and MGd (r = 0.226, *P* = 0.016) in moderate-to-severe DED patients, but not significantly correlated with OSDI score (r = 0.016, *P* = 0.912), TMH (r = −0.002, *P* = 0.988), meibum score (r = −0.196, *P* = 0.317), LLT (r = 0.114, *P* = 0.346), PB (r = 0.116, *P* = 0.338), TB (r = 0.074, *P* = 0.544), meibum score (r = −0.196, *P* = 0.317) and Schirmer I test (r = 0.028, *P* = 0.821).

**Conclusion:**

The novel pressure measurement device exhibited good repeatability and safety in measuring eyelid pressure. Significant correlations were noted between the eyelid pressure and PBR, FBUT, CFS, lid margin abnormality and MGd in moderate-to-severe DED. The measurement of eyelid pressure combined with ocular surface parameters may be valuable for the assessment of DED.

## Introduction

Dry eye disease (DED) has become a common health problem worldwide, which significantly influences the life quality of the patients (1). In 2017, the Tear Film and Ocular Surface Society (TFOS) Dry Eye Workshop (DEWS) updated the definition of DED as “a multifactorial disease of the ocular surface characterized by a loss of homeostasis of the tear film, and accompanied by ocular symptoms, in which tear film instability and hyperosmolarity, ocular surface inflammation and damage, and neurosensory abnormalities play etiological roles.”(2). DED can cause a variety of ocular symptoms, such as dryness, burning and foreign body sensation, and even visual impairment in more severe cases (3). As a chronic ocular surface disease, DED not only affects the quality of life, but even causes harm to mental health (1). Therefore, it is very important to take early treatment and prevent the occurrence of DED. While there are many causes of DED, such as environmental changes, medication use, local inflammation, poor eye usage habits and so on. The friction between the eyelid and the eyeball during blinking and eye movement can also cause the occurrence of DED symptoms (4).

As an important eye appendage, the eyelid exerts a protective effect on the ocular surface. Normal anatomical structure and function of the eyelid can maintain its normal closure, reduce the evaporation of tears, and maintain the stability of the tear film. Blinking and eyelid dynamics play important roles in the distribution of tears and in the maintenance of the integrity of the ocular surface (5, 6). In 1986, Snella (7) reported that the pressure of the eyelid could alter the shape of the cornea, and the concept of eyelid pressure was initially proposed. During the blinking process, the movements of eyelids will generate friction on the ocular surface. Excessive eyelid pressure or increased blink rate can cause related damages to the cornea and conjunctiva (8). Mathers and Lem (9) used an optical interference microscope to demonstrate that the eyelid produced shear force on the cornea during blinking, resulting in corneal epithelial cell damages and changes. Cher (10) proposed the concept of “blink-related microtrauma” in 2003, which described the presence of ocular surface diseases caused by mechanical friction or lubrication disorders of the eye, such as superior limbic keratoconjunctivitis, filamentary keratitides and contact lens related damage. It seems that when the normal physiological state of eyelid changes, it will not only lose the protective effect on the ocular surface, but also cause damage to the ocular surface.

Tear film is the main refractive surface for light to enter the visual system, which can protect and moisten the ocular surface. Stability of tear film is an important indicator of ocular surface health (2). Therefore, when the stability of tear film is destroyed, it can lead to the occurrence of various ocular surface diseases, DED is one of them. Mechanical damage is one of the major causes affecting the stability of tear film, which can cause premature and rapid evaporation of tear. Barros et al. (11) found that under confocal microscopy, the number of stained corneal epithelial cells increased with the increase of mechanical friction, confirming the relationship between mechanical friction and corneal epithelial injury. In addition, long-term mechanical damage can also induce inflammation and destabilize the tear film (4). Tissues fibrosis caused by chronic inflammation can lead to changes in normal anatomical structure, such as lacrimal atresia, which affects the normal drainage of tear, resulting in epiphora. Meduri et al. (12, 13) found that patients with lacrimal atresia usually had ocular surface inflammatory diseases such as chronic blepharitis or chronic conjunctivitis, and showed significant symptoms and signs of epiphora. After punctoplasty surgery was performed to restore normal anatomical structure, the situation of epiphora was significantly improved. It suggests that mechanical damage and inflammatory reaction can be avoided by restoring normal anatomical structure, and then the stability of tear film can be maintained.

As a common ocular surface disease, DED is characterized by unstable tear film combined with a variety of ocular symptoms. Since increased eyelid pressure can lead to ocular surface damage and affect the tear film stability, eyelid pressure may be a cause of DED. To explore the relationship between eyelid pressure and DED, we developed a novel eyelid pressure measurement device. The aim of this study was to evaluate the repeatability and safety of the device and to investigate the correlations between eyelid pressure and ocular surface parameters of moderate-to-severe DED.

## Materials and Methods

### Subjects

This prospective cross-sectional study was conducted from July 2021 to October 2021 at Beijing Tongren Hospital (< city>Beijing < /city>, China). A total of 70 eyes were collected from 6 men (12 eyes) and 29 women (58 eyes). The subjects had an average age of 57 ± 9 (ranging from 32 to 66 years). The diagnosis of DED was based on the TFOS DEWS II definition and classification report. Any subject with any of the following conditions were excluded: (1) age lower than 18 years or higher than 70 years; (2) presence of eye diseases or systemic diseases involving the eyes; (3) a history of eye surgery or trauma to the eye; (4) with blepharoptosis, blepharospasm and other eyelid diseases that significantly affect eyelid pressure. All subjects provided informed consent for their participation in the study, and the entire process complied with the principles of the Declaration of Helsinki. This study was reviewed and approved by the Ethics Committee of Beijing Tongren hospital and registered in the Chinese Clinical Trial Registry (Clinical Trial Registration No. ChiCTR2100054636).

### Eyelid Pressure Measurement

#### Eyelid Pressure Measurement Device

**Figure 1:**
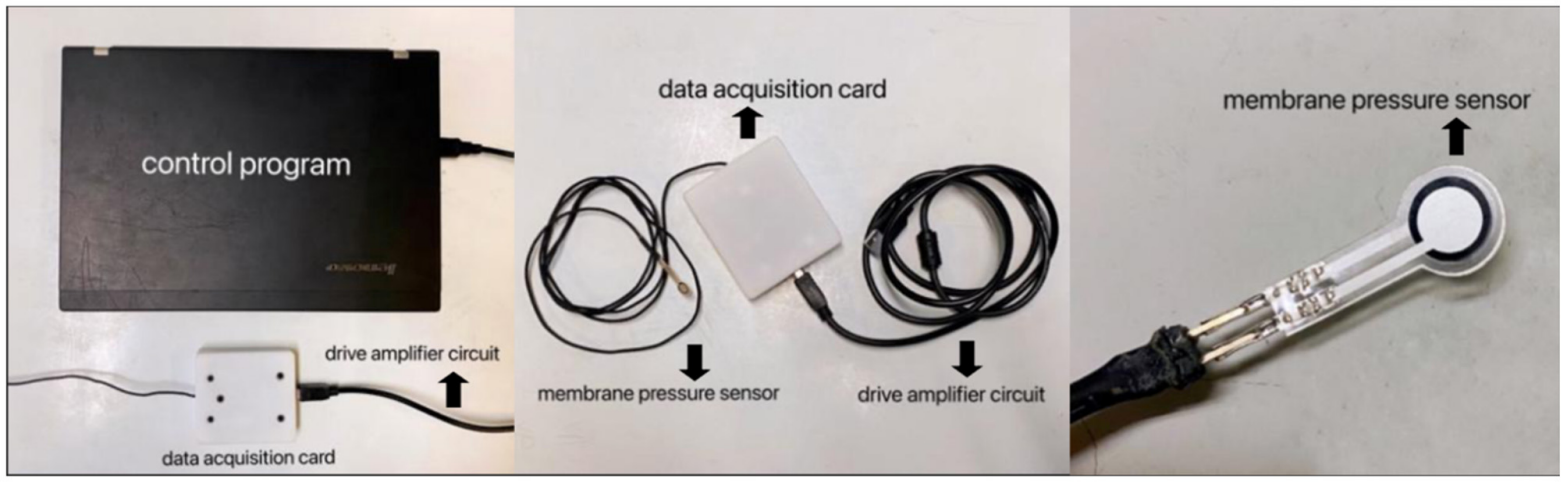
The eyelid pressure measurement device.

The eyelid pressure measurement device was composed of a control program, a data acquisition card, a drive amplifier circuit, and a membrane pressure sensor (Figure 1). Diameter of the sensor was 7.5 mm, the thickness was <0.1 mm, and the measurement range was 0–200 mN. Signal acquisition interval was set to 0.2 ms, and the sampling accuracy was 16 bit. By using a self-developed and designed control program (Labview 2012), the signal changes from the pressure sensor could be continuously recorded. Following setting up, a series of weights with different masses (5 g, 10 g, 20 g) were used to calibrate the system. The weights of different masses were placed on the sensor, and the data collected by the system were recorded, and linearly fit to the pressure in the sensor and the output electrical signal data to obtain the calibration parameters. It seemed that the sensor exhibited very high linearity and could be used for experimental testing.

#### Process of Measurement

**Figure 2:**
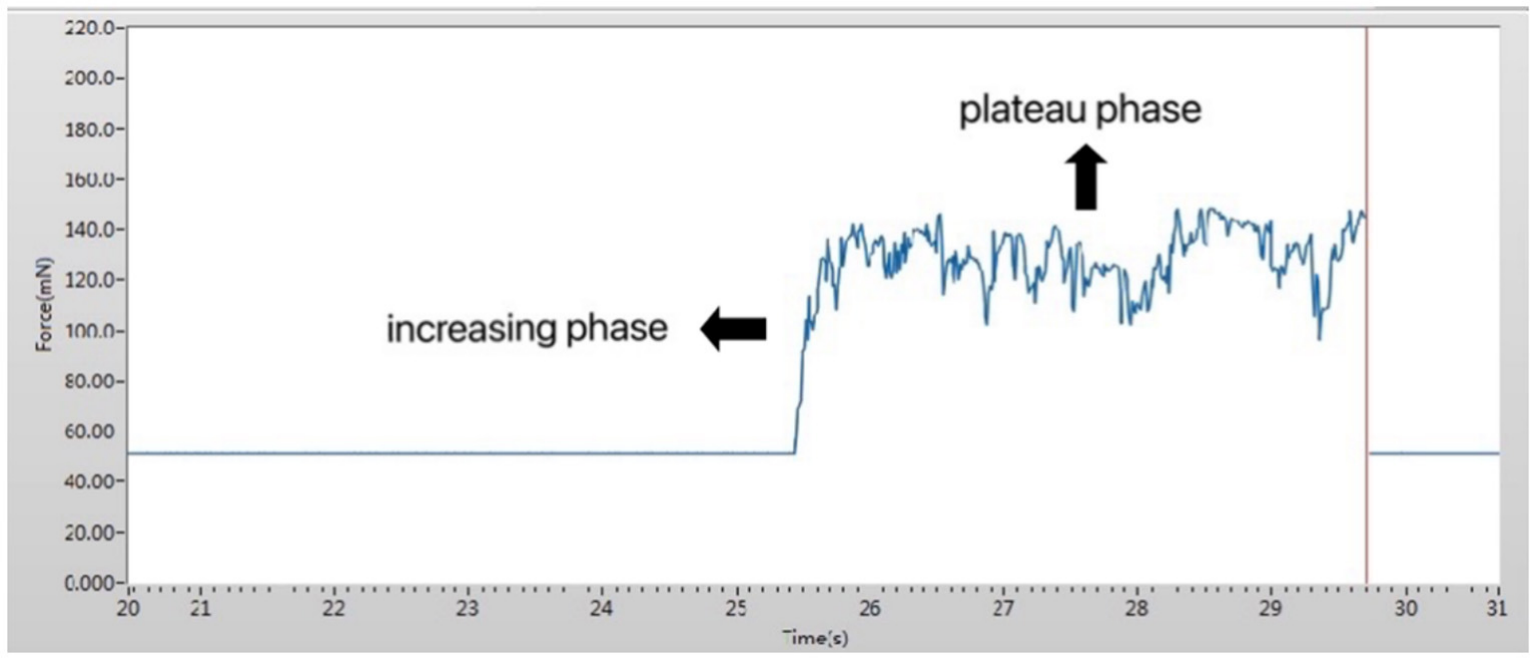
Working interface of the pressure measurement device. The measured pressure was divided into two phases: an increasing phase and a plateau phase.

Upper eyelids pressure was individually measured using the same sensor for each subject. The subject was allowed to sit and topical anesthesia eye drops (Proparacaine Hydrochloride, Alcon, Belgium) were instilled into the conjunctival sac for anesthesia. To protect the cornea, a sterile disposable soft contact lens (Soft Hydrophilic Contact Lens, Horien, China) was placed on the cornea of the subject. A pressure sensor was placed at the center of the upper eyelid. Subsequently, the subject was asked to close his eyes gently without squeezing and retain them closed for 10 sec. The record of the representative eyelid pressure is shown in Figure 2. Eyelid pressure was defined as the average of the values obtained during the 10 sec after the intersection point. After the measurement, one antibiotic eye drop (Chloramphenicol, Shenlong Pharmaceutical, China) was instilled into the patient's conjunctival sac to prevent infection.

#### Repeatability and Safety of the Device

To evaluate the repeatability of the device, an ophthalmologist was trained according to the protocol for the eyelid pressure measurement until proficiency with it. Our measurement was conducted in the same room (temperature 20–25°C and humidity 30–40%) between 10 a.m. and 2 p.m. in a single day. Measurement process was repeated thrice with an interval of no <10 min each time for both eyes of each subject in the same day. To evaluate the safety of the device, conjunctival congestion, secretion production, corneal epithelial injury and other ocular adverse reactions of each subject were observed prior to measurement, 1 and 7 days following measurement.

### Ocular Surface Examinations

#### Process of All Examinations

**Figure 3:**
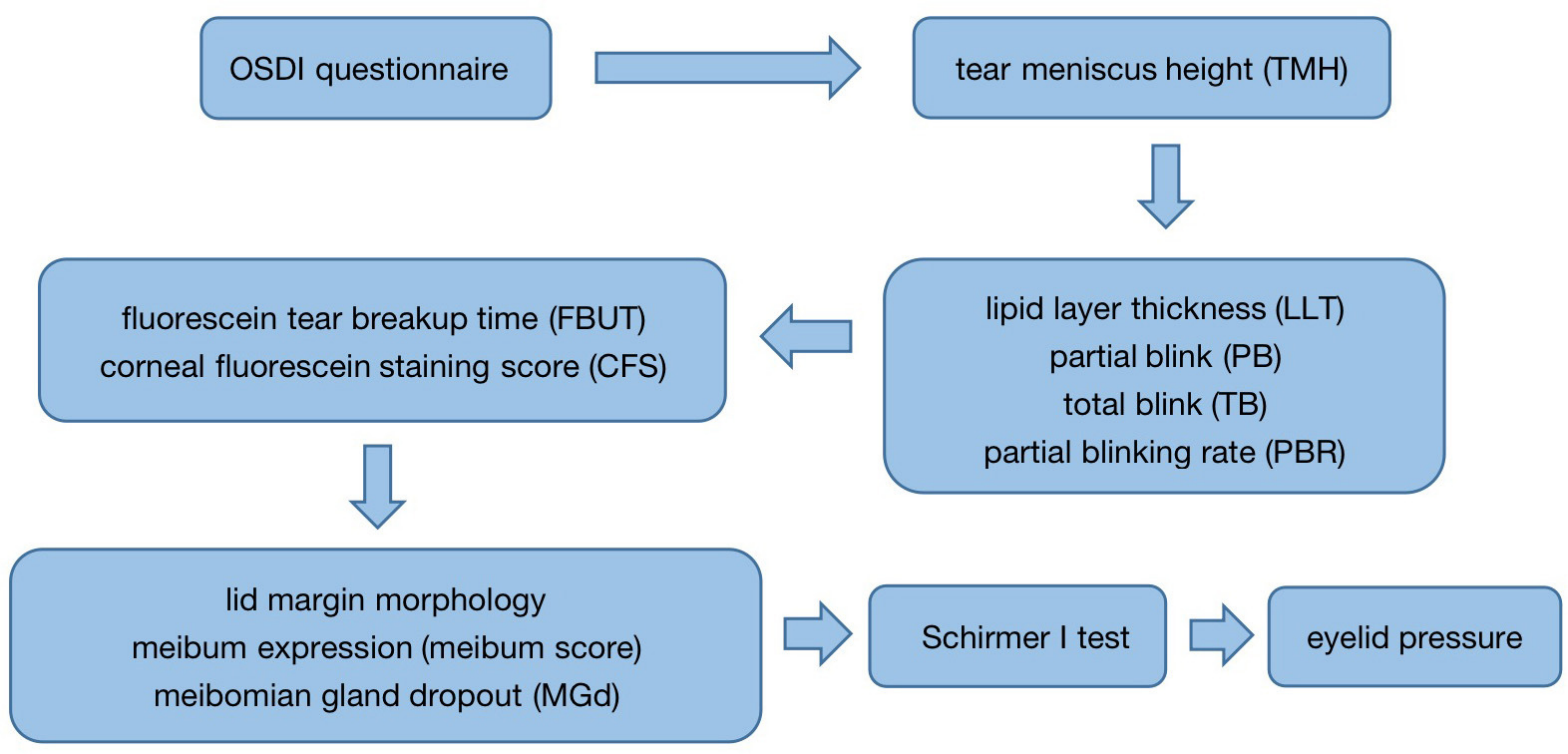
The process of all examinations.

According to DED clinical examination process, all subjects were examined in the following order: first, the completion of the Ocular Surface Disease Index (OSDI) questionnaire was performed prior to all examinations; after that, tear meniscus height (TMH) of the lower eyelid was measured with a Keratograph 5M (K5M) (OCULUS, Wetzlar, Germany); subsequently, the LipiView interferometer (TearScience, Morrisville, USA) was used to measure the lipid layer thickness of the tear film (LLT), the number of partial blink (PB) and total blink (TB) per 20 sec, and the partial blinking rate (PBR); then, 1–2% fluorescein sodium dye was placed into the conjunctival sac of the patient's lower eyelid, and the fluorescein tear breakup time (FBUT) and corneal fluorescein staining score (CFS) were measured; following that, the lid margin morphology and the meibum expression under the slit lamp were evaluated; after that, the images of the upper meibomian glands were obtained to record the situation of meibomian gland dropout (MGd) with the K5M. The subject was allowed to rest for 30 min, and Schirmer I test was performed for 5 min. To avoid the impact on the ocular surface following measurement, eyelid pressure was determined in the end (Figure 3). It had to be emphasized that the entire examination in the process of our study was performed by one examiner.

#### Evaluation of Ocular Surface Parameters

ODSI questionnaire consisted of 12 questions and the score ranged from 0 (no symptoms) to 100 (severe symptoms) (3). TMH was acquired by the measuring tool and recorded in millimeters (mm), based on the image photographed under white light in K5M. LLT, PB, TB and PBR were analyzed by LipiView interferometer based on a 20 sec video. FBUT of each eye was evaluated three times, and the average of three measurements was taken as the final result. CFS was scored according to American National Eye Institute/Industry (NEI) scale, with a total score of 0–15 (14). Eye lid margin abnormality was assessed with a 4-level scale based on the severity of the signs (grade 0–3) (15). Meibum score was assessed with a 4-level scale based on the secretory capacity and the quality in the 5 glands of central upper eyelids (grade 0–3) (16). MGd was assessed with a 4-level scale based on the images of the upper meibomian glands (grade 0–3) (17). The results of Schirmer I test were obtained from the wetted length of the test strip (18).

### Statistical Analysis

All continuous variables were presented as mean ± standard deviations (SD). All categorical variables were presented as median. ICC was used to analyze the repeatability of the device. Pearson correlation coefficient was used to analyze the correlation between two continuous variables, and Kendall's tau-b correlation coefficient was used to analyze the correlation between continuous and categorical variables. A *P* < 0.05 was considered to indicate statistically significant difference. All analyses were performed with the Statistical Package for Social Sciences (SPSS) version 25.0.

## Results

### Repeatability and Safety of the Device

ICC of the measurement results in our study was 0.86. No abnormalities in conjunctival congestion, secretion production, corneal epithelial injury and other ocular adverse reactions were presented in all subjects prior to measurement, 1 and 7 days following measurement.

### Descriptive Statistics of the Subjects

Descriptive statistics of the 35 patients with moderate-to-severe DED are shown in Table 1, including age, eyelid pressure, OSDI score, TMH, LLT, PB, TB, PBR, FBUT, CFS, lid margin abnormality, meibum score, MGd and Schirmer I test.

**Table 1:** Descriptive statistics of 35 moderate-to-severe DED patients.

**Parameters**	**Total eyes (*n* = 70)**
Age (year)	57 ± 9
Eyelid pressure (mN)	92.20 ± 15.60
OSDI score	45.47 ± 17.23
TMH (mm)	0.23 ± 0.13
LLT (nm)	79.47 ± 20.40
PB	5.64 ± 4.32
TB	6.37 ± 4.45
PBR	0.89 ± 0.22
FBUT (s)	3.58 ± 0.67
CFS	4.80 ± 1.87
Lid margin abnormality	1.21 ± 0.41
Meibum score	1.25 ± 1.02
MGd	0.96 ± 0.95
Schirmer I test (mm)	5.26 ± 2.10

*OSDI, Ocular Surface Disease Index; TMH, tear meniscus height; LLT, lipid layer thickness; PB, partial blink; TB, total blink; PBR, partial blink rate; FBUT, fluorescein tear breakup time; CFS, corneal fluorescein staining; MGd, meibomian gland dropout*.

### Relationship Between Eyelid Pressure and Ocular Surface Parameters

**Figure 4:**
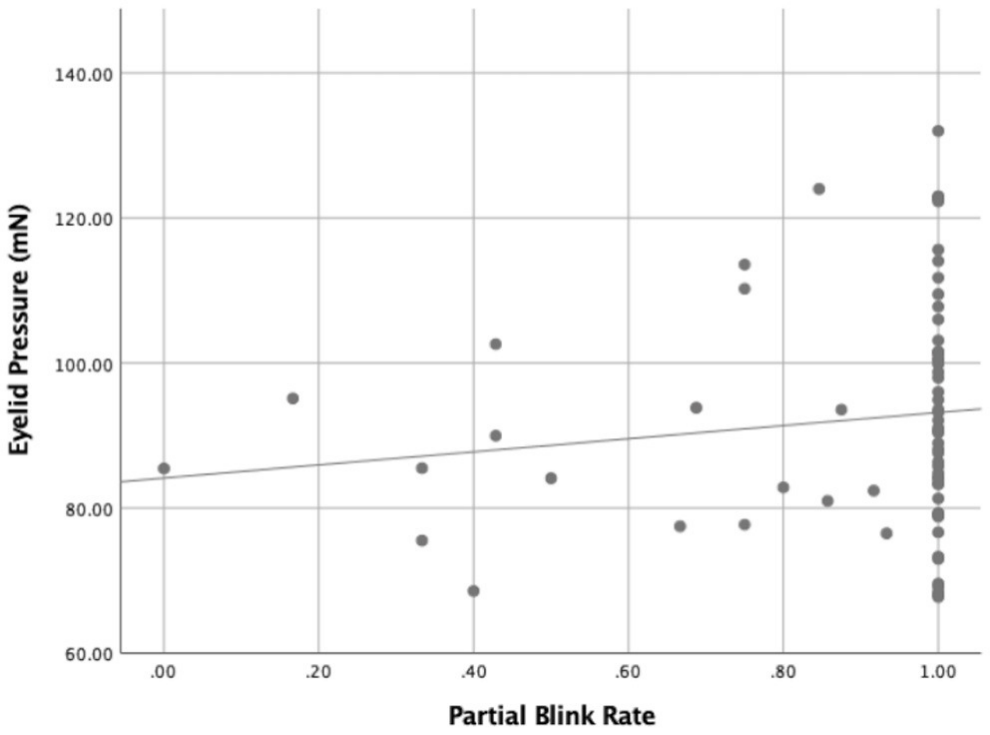
The relationship between the eyelid pressure and the partial blink rate (PBR) (r = 0.286, *P* = 0.016).

**Figure 5:**
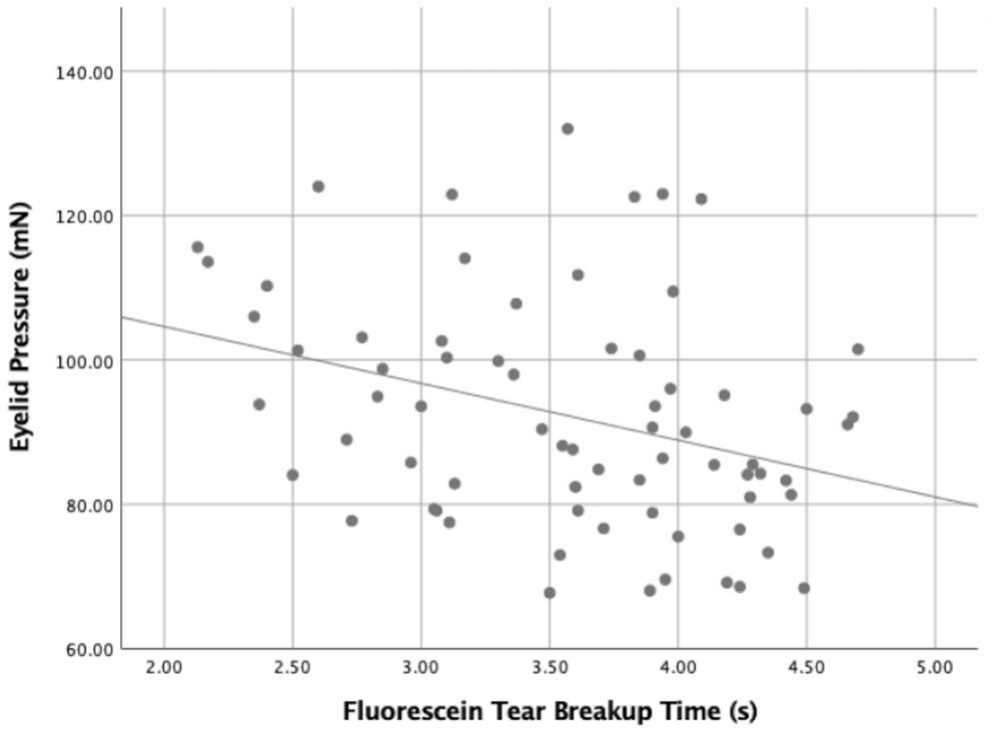
The relationship between the eyelid pressure and the fluorescein tear breakup time (FBUT) (r = −0.331, *P* = 0.005).

**Figure 6:**
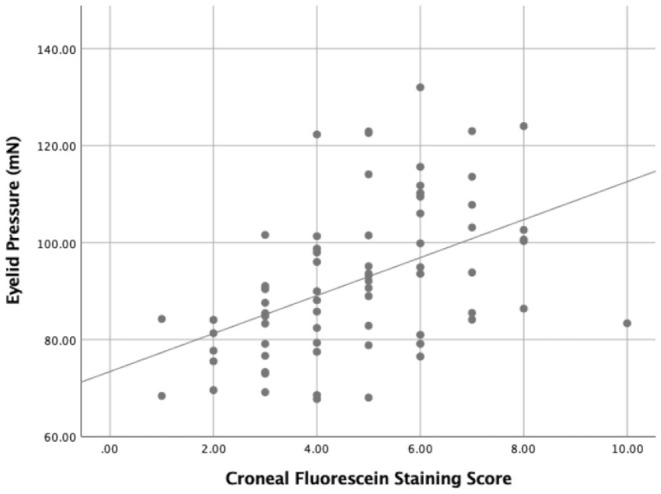
The relationship between the eyelid pressure and the corneal fluorescein staining score (CFS) (r = 0.528, *P* = 0.000).

**Figure 7:**
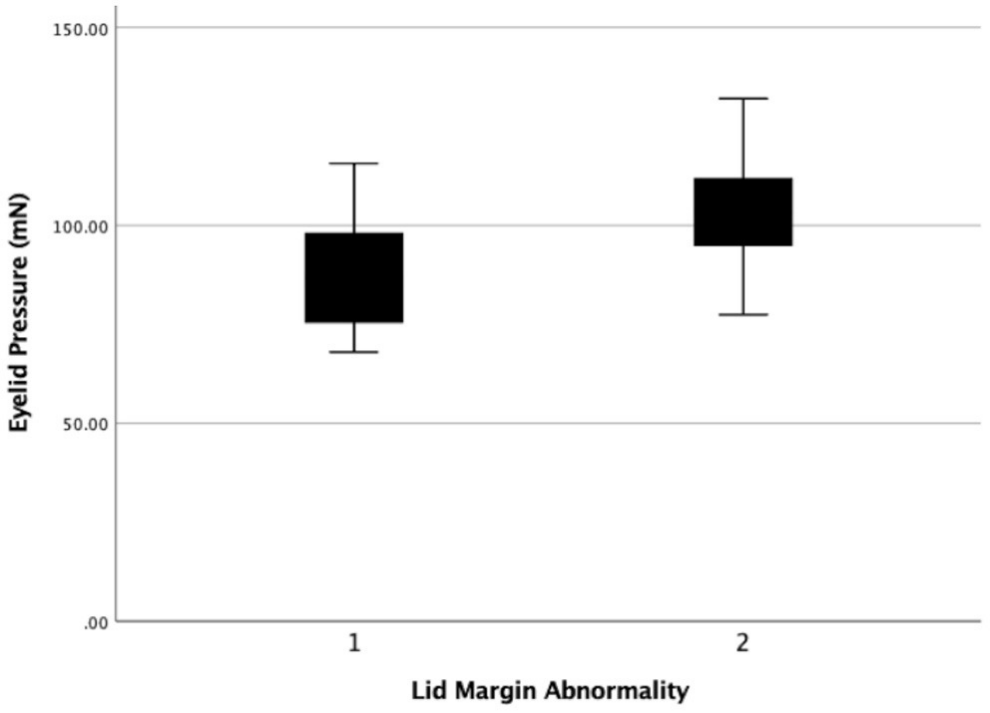
The eyelid pressure of different levels of lid margin abnormality. Statistical significance was assessed between the 2 levels (r = 0.408, *P* = 0.011).

**Figure 8:**
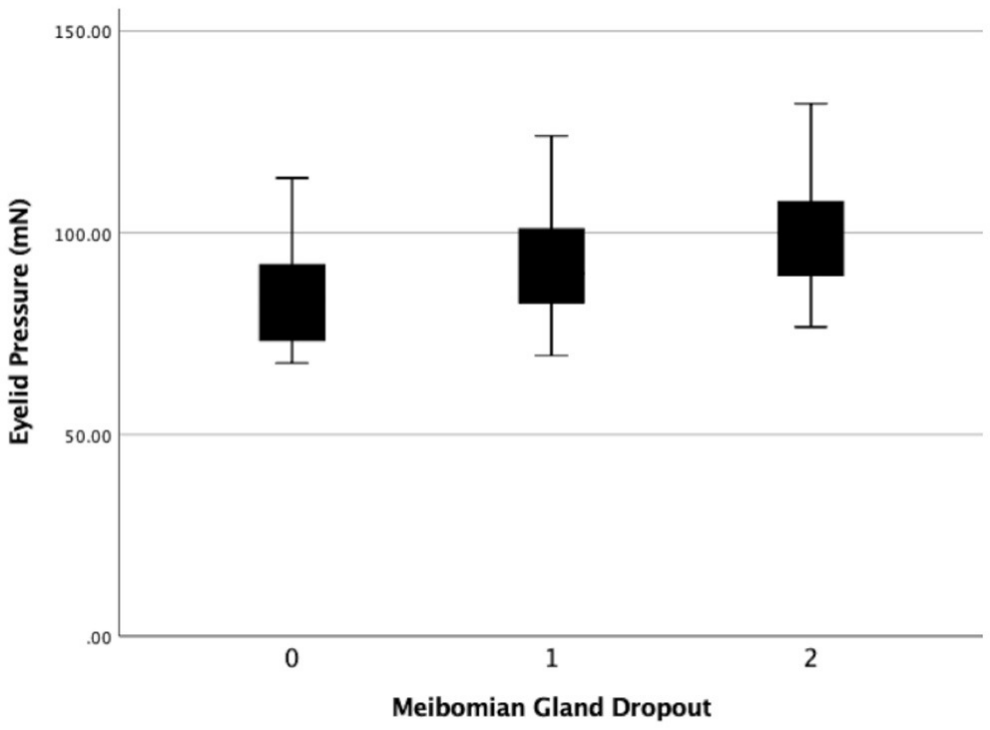
The eyelid pressure of different levels of meibomain gland dropout (MGd). Statistical significance was assessed following comparison of the 3 levels (r = 0.226, *P* = 0.016).

Correlations between the eyelid pressure and ocular surface parameters of moderate-to-severe DED were assessed (Table 2). Upper eyelid pressure was significantly associated with PBR (Figure 4), FBUT (Figure 5), CFS (Figure 6), lid margin abnormality (Figure 7), and MGd (Figure 8). However, no significant correlation was noted between upper eyelid pressure and OSDI score, TMH, LLT, PB, TB, meibum score and Schirmer I test.

**Table 2:** Relationship between eyelid pressure and ocular surface parameters.

**Ocular parameters**	**Eyelid pressure (mN)**	
	**r**	***P*** **-value**
OSDI score	0.016	0.912
TMH (mm)	−0.002	0.988
LLT (nm)	0.114	0.346
PB	0.116	0.338
TB	0.074	0.544
PBR	0.286	0.016^*^
FBUT (s)	−0.331	0.005^**^
CFS	0.528	0.000^**^
Lid margin abnormality	0.408	0.011^*^
Meibum score	−0.196	0.317
MGd	0.226	0.016^*^
Schirmer I (mm)	0.028	0.821

* *p < 0.05;*

** *p < 0.01*.

*OSDI, Ocular Surface Disease Index; TMH, tear meniscus height; LLT, lipid layer thickness; PB, partial blink; TB, total blink; PBR, partial blink rate; FBUT, fluorescein tear breakup time; CFS, corneal fluorescein staining; MGd, meibomian gland dropout*.

## Discussion

Blink and eyelid dynamics play important roles in the distribution of tears and the maintenance of tear film integrity (19, 20). According to TFOS DEWS II, the friction caused by relative movement of the eyelid and eyeball is one of the important reasons for the destruction of the corneal epithelial barrier, which may lead to the development of DED symptoms (4). Several teams have done studies on DED and eyelid dynamics, and found that eyelid pressure in patients with DED was significantly higher than that in normal subjects, and was correlated with ocular surface staining scores and severity of lid-wiper epitheliopathy (LWE) (8, 21–23). The higher the CFS was, the higher the severity of DED became (19). To our knowledge, there were a few studies on the relationship between eyelid pressure and ocular surface parameters in patients with moderate-to-severe DED. Therefore, we selected moderate-to-severe DED patients with corneal epithelial injury to study the relationship between eyelid pressure and ocular surface parameters.

In this study, we developed a novel eyelid pressure measurement device, which was used to measure upper eyelid pressure in both eyes of 35 patients with moderate-to-severe DED three times, and the ICC was used to analyze the results. ICC of the measurement results in our study was 0.86, indicating that the device had good repeatability. No abnormality was noted in conjunctival congestion, secretion production, corneal epithelial injury and other ocular adverse reactions of all subjects prior to and following measurements, indicating that the device was safe for eyelid pressure measurement. We also found that eyelid pressure was significantly correlated with PBR, FBUT, CFS, lid margin abnormality and MGd, but not with OSDI score, TMH, LLT, PB, TB, meibum score and Schirmer I test.

The correlation between eyelid pressure and CFS was similar to the findings by Yoshioka et al. Their study discovered that patients with DED who had higher eyelid pressure showed more serious staining with corneal and conjunctival (24). In addition, they measured eyelid pressure in LWE patients and found that the severity of LWE increased significantly with elevated eyelid pressure (25). With the increase of eyelid pressure, the friction between eyelid and ocular surface during blinking and eye movement also increases, resulting in infiltration mechanical damage of the corresponding parts of the cornea and conjunctival, manifesting as increased CFS. At the same time, the increase of ocular surface friction leads to a series of inflammation, and increase of CFS (4). Although there have been many studies on CFS and FBUT in patients with DED, few studies on the relationship between eyelid pressure and FBUT at present are reported. We believed that the strong correlation between eyelid pressure and FBUT might be because the increase of eyelid pressure led to the enhancement of eyelid friction against the ocular surface, aggravating the instability of tear film and leading to the decline of FBUT.

Meibomian gland dysfunction (MGD) is the main cause of evaporative DED, and MGd, lid margin abnormality, and meibum are the main parameters to evaluate the Meibomian gland function (26). This study found that eyelid pressure was positively correlated with MGd and lid margin abnormality. Chronic high eyelid pressure can cause chronic mechanical irritation to the ocular surface, triggering a chain of inflammatory reactions (4). Atrophy or even loss of the meibomian gland may derive from chronic secretory difficulties and meibomian gland opening obstruction (27). The atrophy and loss of meibomian gland lead to the further reduction of meibum secretion. In order to increase the secretion of meibomian gland, orbicularis muscle will exert more extrusion pressure, which may lead to a corresponding increase in eyelid pressure (26). The increase of eyelid pressure leads to a further enhanced friction on the ocular surface, forming a vicious circle and aggravating the severity of DED.

In this study, eyelid pressure was significantly correlated with PBR. Wang et al. (28) found that an increase in incomplete blinks was positively associated with the risk of DED. Jie et al. (29) further confirmed that incomplete blink was associated with a higher OSDI score, more MGd, and decreased tear film stability. Compared with complete blink, the upper and lower eyelids do not fully contact during incomplete blink, resulting in uneven tear redistribution and aggravating the instability of tear film. Since the friction between the eyelid and ocular surface is related to the eyelid pressure and the contact area between the eyelid and the ocular surface, the higher the eyelid pressure is, the greater the friction is when the corneal area is assumed to be roughly the same (25). We hypothesized that due to the increase in friction, blinking patterns might be more prone to incomplete blink that required less eyelid power, manifesting as increased PBR. PB and TB were recorded in the blink frequency of subjects' normal gaze in the 20 sec video. Due to the influence of visual fatigue, task difficulty and other factors, there was a large individual difference in the blink mode (30). Therefore, we speculated that this was the reason why eyelid pressure had little correlation with PB and TB.

There were still some limitations to our study. First, due to the large size of the membrane pressure sensor, it could not be completely covered by the lower eyelid when put into the lower conjunctival sac, so it was difficult to accurately measure the eyelid pressure of the lower eyelid. Secondly, the pressure was not measured at multiple positions of the eyelid, so we could not get the correlation between the pressure and the staining condition in the corresponding area. Finally, we had limited number of subjects and the results needed to be further confirmed in studies with a large sample size.

## Conclusion

We developed a novel pressure measurement device, which was used to measure the eyelid pressure in patients with moderate-to-severe DED, and analyzed the relationship between the eyelid pressure and ocular surface parameters. The device demonstrated good repeatability and safety. Furthermore, it was found that eyelid pressure in moderate-to-severe DED patients was significantly correlated with PBR, FBUT, CFS, lid margin abnormality and MGd, but not significantly correlated with OSDI score, TMH, LLT, PB, TB, meibum score and Schirmer I test. We believed that the measurement of eyelid pressure might be of important significance for the evaluation of DED, showing higher predictive value in combination with ocular surface parameters of DED.

## Data Availability Statement

The original contributions presented in the study are included in the article/supplementary material, further inquiries can be directed to the corresponding authors.

## Ethics Statement

The studies involving human participants were reviewed and approved by Ethics Committee of Beijing Tongren Hospital. The patients/participants provided their written informed consent to participate in this study.

## Disclosure

This manuscript has not been previously published, and all authors concur with the submission.

## Author Contributions

JW and JB performed the experiment, collected and recorded the data, and prepared the first draft. WS, SL, and YH contributed significantly to the data analysis and case collection. YJ and LT contributed to the concept of the study, helped supervise the project, and gave critical revision of the article. All authors contributed to the article and approved the submitted version.

## Funding

This research was supported by “The Youth Beijing Scholars program”, Natural Science Foundation of China (821704052 and 8217040515).

## Conflict of Interest

The authors declare that the research was conducted in the absence of any commercial or financial relationships that could be construed as a potential conflict of interest.

## Publisher's Note

All claims expressed in this article are solely those of the authors and do not necessarily represent those of their affiliated organizations, or those of the publisher, the editors and the reviewers. Any product that may be evaluated in this article, or claim that may be made by its manufacturer, is not guaranteed or endorsed by the publisher.
